# Of the Calx Muriata

**Published:** 1802-01

**Authors:** W. Simmons

**Affiliations:** Manchester


### *Of the Calx Muriata.*

The Calx Muriata has been lately announced as a remedy for fcrofula, by Dr. Beddoes. In a difeafe in which, in feveral of its forms, the power of art has been fo often baffled, any propofal from authority for averting its deftructive ravages, is entitled to regard. I wifh that I could now congratulate the public on obtaining fo valuable an acquifition, but am forry to ftate that in my own trials of it, it has completely failed.

Scrofula is a difeafe fo common in this town and neighbourhood, that a practitioner connected with an infirmary or difpenfary can feldom be without cafes of it under his care. From my own lift I have felected a fufficient number in the ftate of tumour, of abfcefs, and of ulcer, and in none of them did did it manifeft activity; the afpect of the ulcer was not changed, the tumour difcuffed, nor the abforption of matter promoted. A young man took a tea-fpoon full of the folution, three times a day, in a cup full of weak bitter infufion, for glandular enlargements under the chin, and ulcers in his left leg, without any perceptible alteration in either. A young woman took a tea-fpoon full twice a day, for more than a month, for an ulcerated fub-maxillary gland, which was corroding away fpontaneoufly, without the fmalleft benefit. In thofe collections of fcrofulous matter on the tarfus, which with us ordinarily denote a difeafe in the bones underneath, it has likewife failed. The preparation was obtained from Mr. W. Henry, fo that it could not have been fophifticated. The only fenfible effect of the calx muriata which I have obferved, was that of a gentle laxative.

The cafe of Mifs Johnes, recorded by Dr. Beddoes, is unqueftionably ftrong; but I apprehend it may receive a different interpretation from the one which he has given. At the time fhe began to take the calx muriata there can be no doubt of the nature of her complaint, which was certainly fcrofula; neverthelefs it muft be obferved, that only a fhort time before fhe had fuffered an attack of pleurify, to which and not to the calx, my own difappointments have led me to afcribe her recovery. The ftate of the fyftem under general inflammation, is very oppofite to that peculiar debility incident to fcrofula; in the one, depletion muft be vigoroufly purfued; in the other, remedies calculated to add tone to the fyftem, have been found moft efficacious. This tonic ftate of the fyftem was here produced in a high degree by the pleurify, and thus the accidental acute difeafe, proved the cure of the one which had become almoft habitual. I might ftill further corroborate this opinion, by recalling to the attention inftances of the removal of chronic weaknefs by the fupervention of fever, of which the practice of every one will furnifh fome; and it will alfo be remembered, that one argument in favour of the vaccine inoculation, by feveral of its early advocates, was the advantage which might accrue from employing it with that intention.

In the fenfe in which mercury is a cure for fyphilis, no remedy, I fear, is yet known for fcrofula; fea-air and fea-bathing approach nearer to that character than any other; and in the general form of the difeafe without hectic, as well as for fome particular fymptoms, they are extremely beneficial. A want of vigour is fo palpable, that any general mode of evacuation is feldom proper, but where the head is much affected, as in ophthalmia, leeching may precede the ufe of blifters to the neck or behind the ears. For the fame reafon purging is feldom ufeful, though it is always neceffary to obviate coftivenefs. The topics in ophthalmia confift for the moft part of aftringents; to thefe may be added the vinum opii, a few drops of which inftilled into the eye once or twice a day, is often of fingular fervice. Where the ophthalmia depends chiefly on irritability, a long courfe of the Peruvian bark, and daily ablution of the head with cold water, will materially ferve the patient. And, in order to prevent a return, my experience furnifhes nothing fo effectual, as fhaving the head once a week, and daily wafhing it with cold water. When the patient cannot avail himfelf of the advantages of a refidence near the fea, the glandular fwellings in the neck will be beft difcuffed by a long continuance of the bark, which is alfo ferviceable in the ulcerated form of fcrofula, in which, when fituated upon any of the extremities, dreffing with red precipitate as often as the ulcer becomes indolent or ftationary, will materially affift the cure, efpecially if adhefive plafters be applied in the manner propofed by Mr. Baynton in ulcerated legs.

Scrofula feizing on any of the larger joints, is known by the appellation of white fwelling, which is a frequent caufe of amputation at our infirmary. In the advanced ftage of this diforder, there can be little hope of arrefting its deftructive progrefs by any means; but, where the patient has applied early, a fucceffion of blifters, and healing with the faturnine lotion, have obtained a preference in my own practice over every other mode of topical treatment. Neverthelefs, this muft not be underftood as fuperfeding fuch internal remedies as may be indicated, and among thefe the occafional ufe of emetics will not be neglected.

*Manchester, Dec.* 8, 1801. W. SIMMONS.

